# Hygric Properties of Machine-Made, Historic Clay Bricks from North-Eastern Poland (Former East Prussia): Characterization and Specification for Replacement Materials

**DOI:** 10.3390/ma14216706

**Published:** 2021-11-07

**Authors:** Maria Tunkiewicz, Joanna Misiewicz, Pawel Sikora, Sang-Yeop Chung

**Affiliations:** 1 Faculty of Geoengineering, Institute of Geodesy and Civil Engineering, University of Warmia and Mazury in Olsztyn, Jana Heweliusza 4, 10-724 Olsztyn, Poland; maria.tunkiewicz@uwm.edu.pl; 2 Faculty of Civil and Environmental Engineering, Technical University of Gdańsk, ul. Narutowicza 11/12, 80-233 Gdańsk, Poland; joamisi1@student.pg.edu.pl; 3 Faculty of Civil and Environmental Engineering, West Pomeranian University of Technology in Szczecin, Poland al. Piastow 50a, 70-311 Szczecin, Poland; pawel.sikora@zut.edu.pl; 4 Department of Civil and Environmental Engineering, Sejong University, 209 Neungdong-ro, Gwangjin-gu, Seoul 05006, Korea

**Keywords:** historical materials, clay bricks, resistance to freeze-thaw cycles, compressive strength, MIP, micro-CT, sorptivity

## Abstract

This paper deals with the hygric characterization of early 20th century machine-made clay bricks, representative of great number of historical buildings in north-eastern Poland. Heritage buildings have a high potential for adaptive reuse, which is strictly connected with an urge for knowledge about the properties of these existing building envelopes. To better understand the hygric behavior of historic buildings, various experimental laboratory tests, including density, water absorption, compressive strength and freeze-thaw resistance, were conducted. In order to assess the microstructural characteristics of the tested bricks, mercury intrusion porosimetry (MIP) and X-ray micro-computed tomography (micro-CT) tests were performed. These tests were conducted on clay bricks from historic buildings, as well as on those that are currently being produced, in order to identify the relationship between the materials used in the past and the replacements produced presently. This paper addresses the lack of systematic application of existing standards for evaluating the state of the conservation of historic bricks and for establishing the specifications for replacement bricks. The results of conducted study and further research will be the basis for creating a historic materials database. It would be a useful tool for selecting bricks that correspond with the historically used materials and help to maintain homogenous structure of the restored buildings.

## 1. Introduction

Brick-structured historic buildings are most common in the northern part of Europe, including Poland, and are a significant part of the national heritage. As such, great attention is paid to their conservation and the replacement materials that are used in restoration processes. Undeniably, the main demand made on these building materials is in regard to their durability, which is primarily dependent on the textural and microstructural characteristics of the materials used, their hygric behavior, and their strength. To perform any conservation or restoration processes, especially in terms of replacing the damaged fragments of a wall, a detailed characterization of the historic material is needed [1,2,3,4]. In the north-east of Poland (former East Prussia), a great number of early 20th century historic buildings were erected using machine-made bricks. This technology was widely used in the early years of the previous century, gradually being replaced by modern materials along with the development of reinforced concrete technology and other masonry materials (e.g., autoclaved aerated concrete, silicate blocks, etc.). During their service life (often >100 years), the above-mentioned masonry structures can be subject to decay due to environmental attacks, aging, or damage from long-term heavy loads [5]. There is therefore a lot of interest in preserving historic substances and extending the service life of existing buildings, without substantial alterations regarding the technological and material-wise aspects of reconstruction. The characteristics of materials connected with the presence of water, such as their strength/expansion, resistance to external conditions, as well as their capacity to accumulate moisture within a porous system, are some of the basic parameters influencing their consistent durability and their use in masonry [6]. As mentioned in [7], moisture is known to be one of the main sources of damage to building envelopes. While the literature regarding the hygric properties of both currently produced bricks and historic ones is relatively rich [8,9,10], only a few authors indicate specific replacement materials that might be used in conservation processes.

The direct resistance of clay bricks to freeze–thaw cycles have been studied by researchers in regard to various properties. These include changes in surface appearance [11,12], compressive strength, or the propagation speed of ultrasonic waves through specimens [13], weight [4], and pore structure [14,15,16]. The results of these studies indicate that after freeze–thaw cycles, the surfaces of bricks become damaged, their compressive strength is reduced, and the propagation speed of ultrasonic waves through them is lower. Each freeze–thaw cycle also entails the appearance of new micropores and cracks.

One of the main parameters responsible for permanent frost resistance is the distribution of pores [14,15,16]. Frost resistance prediction models in the literature have focused mainly on pores with a size greater than three microns (the presence of which does not cause frost damage) [17]. However, in many studies, the range of pore sizes responsible for frost resistance is often divergent or completely mutually exclusive. The results of studies on harmful pores often lack consistency and systematicity. In [18], the harmful pores are those below 1.4 μm, while in [19] it is pores below 1.0 μm, in [20] those below 0.74 μm and 0.5 μm, and in [21] those below 0.2 μm. Furthermore, there are also studies [22] in which there is no noticeable relationship between porosity and the frost resistance of masonry elements. Distributions due to structural changes over time may mistakenly indicate that a material has freeze/thaw resistance characteristics. In addition, replacing materials with those with small-diameter pores can also cause rapid frost damage. In reference to manufacturing methods, the authors of [19] and [23] indicate that handmade bricks have a higher content of large pores than machine-made bricks, which might suggest that handmade bricks are more resistant to freeze–thaw cycles. However, there is a lack of knowledge about machine-made historic bricks in the literature. Analyses of the principal component of historic walls and bricks are the best source of knowledge about historic ceramic building materials and assist in indicating the best solutions in conservation procedures [24].

This paper examines the properties of historical bricks, produced by the use of a mechanical brick press, and currently produced restoration, handmade, and factory-made bricks, in order to identify the relationships between the hygric properties of these three types of bricks. In order to determine the compatibility between the materials used in the past and their presently produced replacements, comparative analyses using different techniques (including compressive strength, freeze-thaw resistance, and water sorptivity) were conducted. Mercury intrusion porosimetry (MIP) and X-ray micro-computed tomography (micro-CT) tests were performed to assess the microstructural characteristics of the tested bricks. Afterwards, a systematic comparison of the data obtained with the recommendations in the existing literature is presented, thereby determining the suitability of modern restoration materials.

## 2. Materials and Methods

### 2.1. Description of Materials

**Figure 1:**
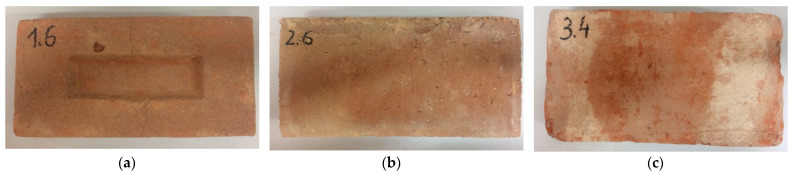
Samples of bricks: (**a**) hand-made, (**b**) factory-made, and (**c**) historic.

The materials studied consist of three types of bricks: historic bricks (machine-made) and two currently produced restoration bricks, namely handmade bricks and factory-made bricks. Both the handmade bricks (Figure 1a) as well as the factory-made (Figure 1b) bricks were sampled from local brickyards, while the historic bricks (Figure 1c) were carefully sampled from the demolished fragments of a building of the former garrison jail on Artyleryjska Street (Olsztyn, Poland). 

**Figure 2:**
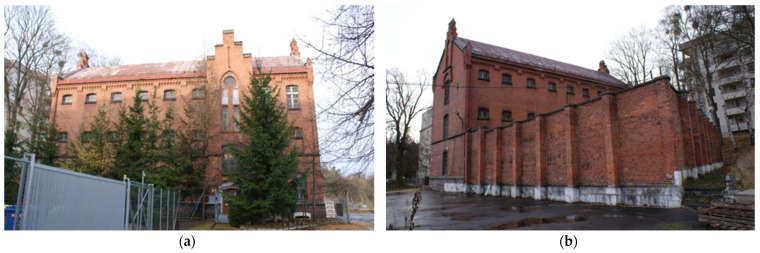
Building of the garrison jail: (**a**) from the south, (**b**) from the north-eastern with the perimeter wall encircling the prison yard.

The building was erected between 1899 and 1900 on the north-eastern side of this historic military complex. The longitudinal axis of the three-story building, with a partial basement underneath, was laid in an east-west direction. The monumental neo-gothic style clearly refers to fortified architecture. For over 100 years, the building served as a military jail, as a part of The Military Property Agency estate, with a military court in its western part and prison-cells in its eastern part. The building is currently being renovated (Figure 2) [25].

The brickyard signature found on the bricks suggests that they were made in Drulity (Poland) using the Schlickeysen brick press. The historic brick samples were measured with an electronic caliper. The comparative material produced nowadays (hand-made and factory-made bricks) was selected on the basis of the physical and mechanical properties specified by the producers.

### 2.2. Description of Testing Methods

#### 2.2.1. Compressive Strength and Freezing and Thawing Resistance

The direct resistance of bricks to freeze-thaw cycles was tested according to the PN–B–12012:2007 standard [26], on the three above-mentioned types of bricks. Each series was comprised of two sets of bricks (one set = ten bricks). All of the samples were collected in accordance with EN 771-1 [27]. According to the chosen standard, the samples were saturated in water for 48 hours and exposed to temperatures of –15 ± 2 °C for four hours in a climate chamber (Uni-mors, Poland). The temperature stability in the frost resistance test chamber was +/−1 °C, with a temperature range from 30 °C to +30 °C. The device used enables testing according to the selected standards and procedures. The samples were subsequently submerged in water for four hours. For facade elements, this cycle was repeated 25 times. After testing, a brick in any of the examined samples was considered durable to freeze-thaw cycles if the number and size of the edge, angle damage and surface cracking was lower than what is specified in PN-B-12012:2007, after 25 freezing cycles followed by defrosting in water. After being exposed to the freeze-thaw cycles, the compressive strength of the bricks was determined and the results were compared to the compressive strengths of bricks form the same series, which had not been exposed to the freeze-thaw cycles. In this way, a ratio between compressive strengths before and after the freeze-thaw cycles was acquired, as a quantitative indicator of brick resistance to freeze-thaw behavior. Compressive strength, before and after freezing, was measured on ten brick samples taken from each type of brick, according to EN 772-1+A1:2015−10 [28]. Before testing, the samples were cut out from the bricks. Rollers, with a diameter of 100 mm and a height of 60 mm, were prepared for the historic bricks, while cuboids were prepared for the factory-made bricks (100 mm × 100 mm × 60 mm) and hand-formed bricks (100 mm × 100 mm × 53 mm). The surfaces of all of the testing units were prepared by grinding, to obtain the flat surface required by EN 772-1+A1:2015–10. The samples were subsequently dried to a constant weight at 105 ± 5 °C. 

#### 2.2.2. Mercury Intrusion Porosimetry (MIP)

Pore size and the space between pores are two brick properties considerably affected their durability [29,30]. Assessing these parameters is crucial in establishing the specifications for replacement bricks. The porosity of the tested bricks was determined with an AutoPore IV 9500 mercury porosimeter (Micromeritics, Atlanta, GA, USA). Three test samples were taken from each type of brick. The samples, drilled from the parent material, were cylindrical in shape (approximately 13.0 mm in diameter and 17.0 mm in height). Dust was removed from the surface of the samples using compressed air. Before testing, the samples were dried to a constant weight. The porosity distribution was determined during the test, using a working pressure of up to 33,000 Psi. Penetrometers, with a measuring vessel capacity of 3 cc and 0.4 cc and in different capillaries, were used for the tests. The study allowed the determination of porosity structure, pore distribution, mean pore diameter, and total porosity. Pores ranging from 300.00 μm to 0.006 μm were distinguished.

#### 2.2.3. X-ray Micro-Computed Tomography (Micro-CT)

In addition to the MIP measurements, micro-CT was also conducted to evaluate the pore structures in detail. Micro-CT is a nondestructive approach which allows investigation of the inner structure of a target material without destroying it. The method has been used widely to examine the characteristics of construction materials [31,32,33]. Although pores of less than a few orders of micrometers can be measured using MIP, pores larger than those in this range also strongly affect material properties such as compressive strength and durability. As such, pore characteristics with relatively large sizes also need to be considered. 

**Figure 3:**
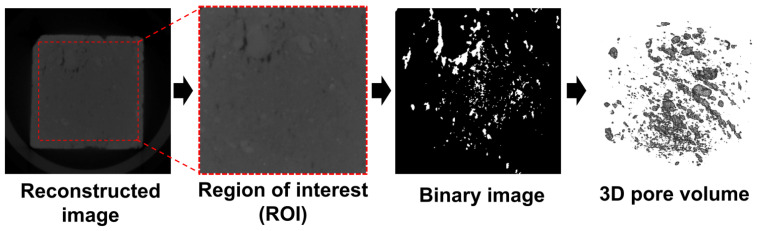
Micro-CT imaging procedure for classifying pores in a target specimen and for visualizing them in 3D.

Figure 3 shows the micro-CT imaging procedure used with the materials in this study. A SkyScan 1173 (Bruker, Billerica, MA, USA) was used for the micro-CT measurements, with the measurement conditions set at 130 kV and 61 mA. Cubic specimens with a 20 mm edge length were used to produce high-resolution images. Since the main target of the micro-CT measurement was the pore part, imaging was performed to segment the pores from the rest of the specimen. Once a target specimen was scanned, an 8-bit grayscale image could be formed, denoted as the reconstructed image. A region of interest (ROI), composed of 300 × 300 pixels with a size of 46.9 μm, was selected to achieve a more effective investigation. Each 8-bit image pixel was expressed by 256 values (0–255), with the value determined according to the relative density of the phases. A proper threshold needs to be selected in order to segment the pores and a modified Otsu method [34] was used for this purpose. In the binary image, the white represents pores within the sample, while the black is the solid part. A 3D volumetric image can be obtained by consequently stacking the binary images, as shown in the last image in Figure 3. The 3D pore image of each specimen was generated with this procedure, with the pore characteristics of each material, at normal and freeze-thaw states, investigated using the volumetric images.

#### 2.2.4. Water Sorptivity 

Liquid suction is one of the physical phenomena which should be strictly controlled in order to prevent the occurrence of irreversible deterioration processes, such as cracks, delaminations, or soluble salt concentrations [35,36]. The moisture distribution in a specimen is strictly connected with its structure. The pace, as well as the volume, of the absorbed water are derived from the shape, structure, and volume of pores. In the case of specimens with narrow pores, the capillary rise will be high but relatively slow. Conversely, specimens with wide pores will be characterized by low and fast capillary rise. The theoretical equation defining the rate of sorption in relation to the pore radius is the Formula (1):

(1) $$\nu =\frac{r^{2}}{8\eta l}\left(\frac{2\sigma \cos\theta }{r}+gql\cos\vartheta \right)$$

where *ν* is the sorption velocity, *r* the pore radius, *q* the liquid density, *η* the dynamic viscosity of the liquid, *g* the gravitational acceleration, and σ the surface tension. The presented Equation (1) applies to vertical and horizontal transport of liquids in pores and capillaries of the material, where the influence of gravity is relatively small compared to the liquid viscosity forces.

The sorptivity of the brick samples was determined by monitoring the increase in weight of a specimen over time during capillary water absorption. This study was conducted using the direct gravimetric method with all the samples dried to a constant weight at 105 ± 5 °C. Afterwards, the side face of each brick was placed on rods in a tray containing water so that the entire lower surface of the specimen could be in good contact with the water. The samples were weighed at regular intervals as the study was being conducted in order to determine the quantity of liquid absorbed. The sorptivity was determined from the gradient of the plot of the volume of water absorbed (per unit area of inflow surface), against the square root of time. For each specimen, seven points were obtained (with the required minimum being five points) [37].

## 3. Results and Discussion

### 3.1. Sorptivity

Sorptivity studies using the gravimetric method are simple and therefore widely used for moisture determination. This method makes it possible to collect data about the quantity of moisture inside a specimen and to draw preliminary conclusions, in terms of material microstructure, regarding frost resistance. Table 1 shows the results of sorptivity tests for three samples of each type of brick, before the freezing and thawing resistance study. The test results indicate that the handmade brick had similar properties to the historic brick in terms of water transport.

The sorptivity study showed the greatest discrepancy between the historic brick and the factory-made brick, with the sorptivity of the former being more than twice as large as that of the latter. The difference between the historic and hand-made bricks was within 20%.

The sorptivity results indicate that factory-made bricks may have limited frost resistance. The pace of capillary transport in ceramic materials with pores bigger than 0.1 μm is relatively fast, but such pore structures do not lead to frost damage. The pace of water absorption is slower in bricks with pores smaller than 0.1 m, (observed in factory-made bricks), but such materials are more prone to damage due to frost.

### 3.2. Assessment of Frost Resistance 

**Figure 4:**
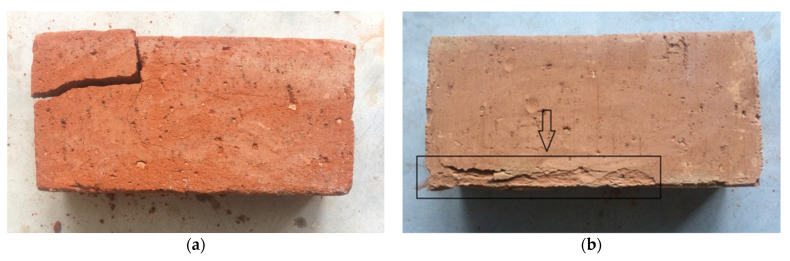
The appearance of non-resistant bricks after the freeze-thaw study: (**a**) complete damage (historic brick), (**b**) damage in the form of delamination (factory-made brick).

Two out of three types, exposed to twenty-five freeze-thaw cycles, were damaged (Figure 4). Some of them were totally destroyed during the study and the others had damage in the form of cracking or delamination (Figure 4b). However, the third type of tested material (hand-made bricks) had no signs of damage during a visual examination. Their structure remained unchanged.

A brick is resistant to freeze-thaw cycles if, after 25 cycles, the number and size of the edge, angle damage, and cracking on the surfaces is lower than specified in PN-B-12012:2007. Taking into account the criteria specified by this standard, only the hand-made bricks were resistant, while the factory-made and historic bricks were not. However, the historic bricks might have been exposed to atmospheric conditions, including freeze-thaw cycles, prior to the study. The compressive strengths of the bricks before and after the freeze-thaw cycles, with the corresponding standard deviation and ratio between the pre- and post-freeze-thaw cycle compressive strengths, are shown in Table 2. They were calculated using the average values of the compressive strength pre- to post-freezing.

### 3.3. Porosity and Pore Size Distribution 

To investigate the pore characteristics of the specimens, the porosity and pore size distributions of the materials under consideration were examined using MIP and micro-CT. As mentioned above, MIP can measure pores smaller than a few micrometers, while micro-CT can be used to examine relatively large pores.

#### 3.3.1. MIP Results

**Figure 5:**
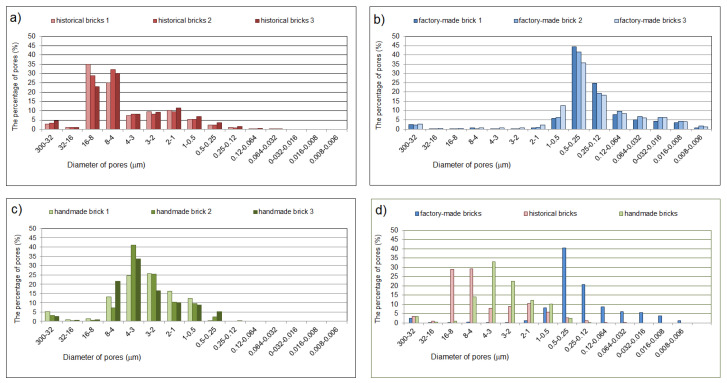
Distribution of pores in brick samples (**a**) historical brick, (**b**) factory-made brick, (**c**) hand-formed brick, and (**d**) a comparison of the average porosity distributions of all bricks.

Figure 5 shows the porosity distributions in the brick samples tested. Three samples of each type of brick were tested.

The pores were divided into ranges according to their size, in reference to the literature. The first range included large pores whose diameter was greater than 3 μm [38,39]. The second range was that of medium pores (1–0.1 μm) and the last one was that of small pores smaller than 0.1 μm [29] (Table 3, Table 4 and Table 5).

The following parameters were additionally determined during porosimetric analysis: total pore area, median pore diameter, bulk density and porosity (Table 5). Mercury porosimetry only makes it possible to determine the total pore area for open pores, ignoring the closed ones. Based on the analysis, the average pore diameter was also calculated, assuming that the pores were perfectly cylindrical in shape.

All brick types had similar porosity and bulk density values (Table 6). The total porosity in all samples oscillated around 30%. The most porous were the hand-made bricks (average 32.03%), while the least porous were the factory-made ones (average 28.89%). Figure 5 shows different pore distributions, depending on the type of sample. Factory-made bricks were characterized by a clear predominance of sizes ranging from 1.0 to 0.12 micrometers (Figure 5b). Handmade brick samples had most pores ranging from 8.0 to 0.5 micrometers (Figure 5c). However, in samples taken from the historic building, the dominant pore volume was in the range of 16.0–4.0 micrometers (Figure 5a). The historical bricks and factory-made bricks had one dominant peak, reaching over 40% of the volume of all the pores. However, in the case of the historic and hand-made bricks, the peak in terms of the porosity distribution was relatively similar (Figure 5d). Bulk density in all of the measured samples was approximately 1.5–1.8 g/mL. Despite having the highest overall porosity, the handmade brick had the lowest density (1.55 g/mL). It is worth noting that although all specimens have similar density, their structure of pores and median pore diameter are different. That indicates for individual characteristic of water absorption (Table 1) for all types of bricks and consequently different freezing and thawing resistance. The sorptivity of the handmade and historic bricks was relatively high (2.0–2.5 $\mathrm{mm}\times \min^{\frac{-1}{2}}$), meaning that the pore diameter was relatively large. The sorptivity of the factory-made brick was low (0.7$\;\mathrm{mm}\times \min^{\frac{-1}{2}}$), indicating for the domination of the small diameter of pores (responsible for frost damage of ceramic materials). In order to determine the applicability of the materials to be used in renovating existing facilities and to determine the frost resistance of a material on the basis of porosity distributions, the tables below show predicted resistance to freezing / thawing based on data from the literature. Table 7, Table 8 and Table 9, presents data separately for each sample as well as a cumulative average for all of them. The last column shows whether the sample was frost-resistant (FR) or non-frost-resistant (NFR).

Table 7, Table 8 and Table 9 (above) show the frost resistance prediction models in relation to pore volume (as a percentage) for the range of interest, in reference to the literature. The researchers in [11] proved that the size of the pores responsible for frost resistance ranges from 1.0 to 10 micrometers. According to [18], porosity in the range from 1.4 to 0.25 micrometers is not dominant. Many authors [15,22,30,40,41] have indicated that a significant number of pores larger than 3.0 micrometers are important for frost resistance. In [19], the researcher observed that materials with a larger pore volume, below 1.0 micrometers, have lower frost resistance than those with a predominance of pores larger than 2.0 micrometers. In [42], it was found that the dominant pores, smaller than 1.0 micrometers, are not responsible for frost damage. However, the article also argues that these pores increase in volume over time and change their character into a destructive one. The greatest risk of frost damage, according to [20], is caused by the presence of pores in the range below 0.5 micrometers, while their dominance, together with pores smaller than 0.75 micrometers, is the main factor in material destruction. The Maage models-DF = 3.2/PV + 2.4 · P3–assume that a material is frost resistant when DF > 70 and not resistant when DF < 70; where PV is the total volume of pores and P3 is the volume of pores with a diameter larger than 3.0 micrometers. Franke and Bentrup’s [43] models are connected with a median pore size of Ф50, with a material being frost resistant when Ф50 ≥ 1.65 μm and not resistant when Ф50 ≤ 0.60 μm. 

#### 3.3.2. Micro-CT Measurement

**Figure 6:**
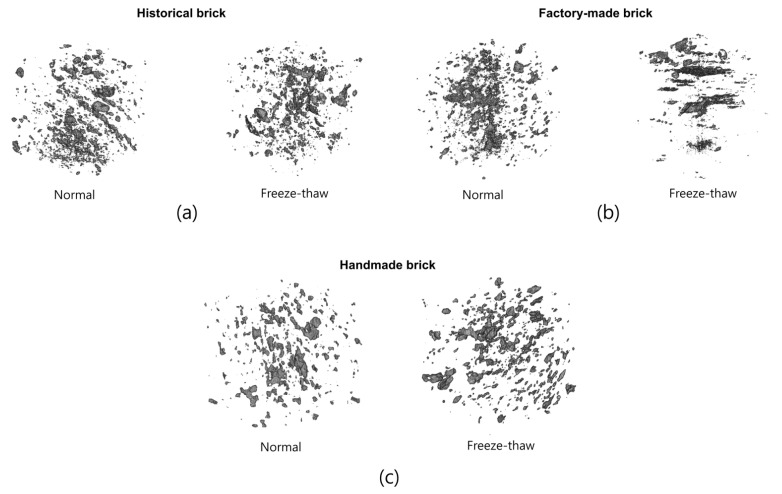
Volumetric pore images of (**a**) historical bricks, (**b)** factory-made bricks, (**c**) handmade bricks (in each case, the left hand side image shows the pore structure in the normal state, while the right hand side image is of the pores after the freeze-thaw cycles).

The relatively large pores were investigated using micro-CT data. Only pores larger than 46.9 μm (which can be denoted as voids) were considered, considering the resolution of the images used (Figure 6). 

Figure 6 shows the pore images of historical, factory-made and handmade bricks, respectively. In this figure, the left hand side image represents the pore structure in a normal state, while the right hand side one shows the pores after the freeze-thaw cycles. It is clear that the pore characteristics of in case are slightly different, according to the specimen type. The historical bricks tended to have relatively large and regular pores, which seems to be similar to the pores of the handmade bricks, while the factory-made bricks contained more tiny and anisotropic pores. In all the cases, no cracks were observed in the internal microstructures. For a quantitative comparison, the porosity of each kind of brick was computed using the 3D pore images. In the normal state, the measured porosity values were 7.8% (historical), 6.3% (factory-made) and 7.5% (handmade). After the freezing-thawing tests, the porosity became 7.9% (historical), 6.4% (factory-made) and 9.6% (handmade). For the relatively large pores, the results confirm that there was no significant change in porosity, although the handmade case showed about a 2% difference. It can be concluded that all the bricks examined were stable in terms of voids for freeze resistance.

**Figure 7:**
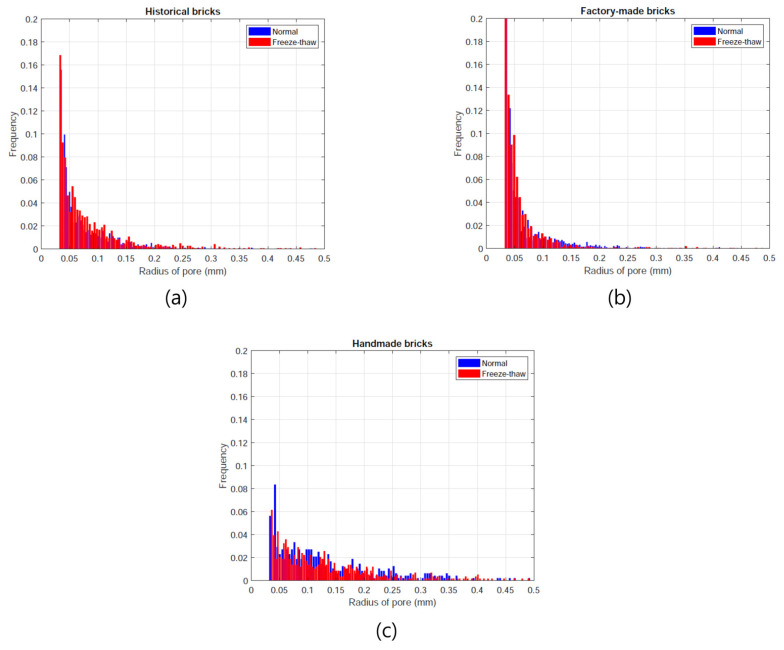
Pore size distributions of each case (**a**) historical bricks, (**b**) factory-made bricks, (**c**) handmade bricks (Note: in each figure, the blue denotes the pore size distribution in the normal state, while the red is the size distribution after freeze-thaw cycles).

For a more detailed analysis, the pore size distribution of the specimens was also examined (Figure 7). In the pore size distribution, only the pores in Figure 6 were taken into consideration; the pore sizes in each case and their differences, before and after the freeze-thaw cycles, can be seen. In all the cases, there was no clear change in the pore size distribution according to the freeze-thaw cycles. This indicates that the materials showed good freezing resistance performance in terms of void distribution. For a comparison of pore size distribution, the historical brick can be used as a reference. In the case of the factory-made brick, there was a much higher proportion of small pores (<0.05 mm) than in the case of the historical brick. As mentioned above, large pores are important in determining frost resistance; a material with more small pores at similar porosity levels can be less effective in enhancing frost-related performance. In contrast, the handmade bricks had a lower portion of small pores and relatively large pores than the reference material, which can be attribute to the advanced frost resistance performance.

The micro-CT results confirm that the general porosity for the large pores was similar in all the cases, but their pore size distributions showed different tendencies. The handmade brick showed a similar pore size trend to the historical brick, which can result in higher frost-resistance properties. However, the factory-made brick had a high number of small pores, which are known to be disadvantageous in terms of hygric properties.

## 4. Conclusions

This paper deals with the hygric characterization of historic clay bricks, manufactured with a brick press, which are representative of early 20th century heritage buildings in north-eastern Poland. The physical and hygric properties experimentally characterized in this work were also compared with the properties of currently produced handmade and factory-made bricks, in order to establish the specifications needed for replacement bricks which are to be used in restoration processes.

As a result of porosity measurements with a mercury porosimeter and micro-CT as well as frost resistance and sorption tests, the following conclusions can be drawn:

- The distribution of porosity in factory-made bricks (mainly the lack of pores larger than 3 micrometers), indicates that these masonry elements cannot be used in renovating historic structures. However, despite the lack of frost resistance (in reference to the industry standard), the brick was still characterized by a high compressive strength of 37.0 MPa, even after 25 cycles of freezing and thawing.
- Almost all of the indicators related to pore distribution support the assumption that hand-formed bricks are appropriate for use in restoration, from the point of view of frost resistance.
- Despite similar porosity and density values, all the types of bricks studied had significant differences in pore structure, as confirmed by the median pore diameter values and sorptivity test results. The factory-made bricks had mainly narrow pores, which are responsible for slow but significant capillary action (resulting in frost damage). The apparent resistance of the historical bricks to freezing and thawing (observed on the basis of their pore distribution) may have been a result of changes in their microstructure and chemical composition over time, but certainly not from their original structure.
- The indicators used to predict frost resistance, including the Maage and Franke and Bantrup factors above all, satisfactorily determine ceramic materials’ resistance to freezing/thawing.
- All of the models presented above, for predicting frost resistance, gave a useful indicator. However, tests on real samples are still the most important.
- The sorptivity study indicated that capillary action in the handmade and historic bricks (although machine-made) were similar, which is crucial when considering water transport within an entire wall. However, the ability of historic bricks to pull water was much lower.
- The comprehensive strengths of the historic and hand-made bricks, before the freeze/thaw study, were very similar. However, the factory-made bricks were characterized by significantly higher comprehensive strength. Comprehensive strength after the freeze/thaw study could not be compared, due to the lack of test results for the historic bricks. However, it can be assumed that it would be similar to that of the handmade brick.

The results of this study clearly indicate that handmade bricks are good replacement materials that can be used in renovation processes, providing a homogeneous structure to historic walls, in terms of hygric characteristics. 

Due to the scarcity of data regarding the hygric behavior of historic bricks, we recommend further research, which would contribute to the creation of a historic materials database, in terms of porosity, pore size distribution and hygric properties. This would be a useful tool for assessing the state of historic bricks and consequently selecting appropriate replacement materials.

In the interest of natural environment many of polish brick manufacturers declare that their products are made without chemical admixtures, dyes or ash additives to make them more ecologic.

## Figures and Tables

**Table 1:** Sorptivity results for particular brick samples.

Bricks Type/Property	Historic Bricks	Factory-Made Bricks	Handmade Bricks
$\mathrm{Sorptivity}\;\mathrm{of}\;\mathrm{sample}\;1\;[\mathrm{mm}\cdot \min^{\frac{-1}{2}}]$	2.1445	0.7471	2.501
$\mathrm{Sorptivity}\;\mathrm{of}\;\mathrm{sample}\;2\;[\mathrm{mm}\cdot \min^{\frac{-1}{2}}$]	2.5256	0.6970	2.4697
$\mathrm{Sorptivity}\;\mathrm{of}\;\mathrm{sample}\;3\;[\mathrm{mm}\cdot \min^{\frac{-1}{2}}]$	2.0354	0.7215	2.0967

**Table 2:** Compressive strength of bricks before and after freezing with the corresponding standard deviation and the ratio of compressive strengths pre- to post-freezing.

Bricks Type/Property	Historical Bricks	Factory-Made Bricks	Handmade Bricks
Compressive strength (MPa)	14.07 ± 2.51	44.04 ± 4.37	14.20 ± 3.87
Compressive strength after exposure to freeze/thaw cycles (MPa)	-	37.01 ± 5.88	11.84 ± 2.26
The attitude of compressive strength after and before exposure to freeze/thaw cycles	-	0.84	0.90

**Table 3:** The pore size ranges of historical bricks.

Sample/Total Volume of Typical Pore Ranges	Historical Brick No. 1	Historical Brick No. 2	Historical Brick No. 3	Average of Historical Bricks
Large-size pores-larger than 3.0 μm [%]	75.14	71.47	73.53	73.38
Medium-size pores-3.0 μm–0.1 μm [%]	24.70	27.41	25.55	25.89
Small-size pores-smaller than 0.1 μm [%]	0.16	1.12	0.92	0.73

**Table 4:** The pore size ranges of factory-made bricks.

Sample/Total Volume of Typical Pore Ranges	Factory-Made Brick No. 1	Factory-Made Brick No. 2	Factory-Made Brick No. 3	Average of Factory-Made Bricks
Large-size pores-larger than 3.0 μm [%]	4.12	3.85	6.14	4.70
Medium-size pores-3.0 μm–0.1 μm [%]	62.11	65.66	66.40	64.72
Small-size pores-smaller than 0.1 μm [%]	33.77	30.49	27.46	30.57

**Table 5:** The pore size ranges of handmade bricks.

Sample/Total Volume of Typical Pore Ranges	Handmade Brick No. 1	Handmade Brick No. 2	Handmade Brick No. 3	Average of Handmade Bricks
Large-size pores-larger than 3.0 μm [%]	90.11	52.35	59.42	67.29
Medium-size pores-3.0 μm–0.1 μm [%]	9.89	47.65	40.33	32.62
Small-size pores-smaller than 0.1 μm [%]	0.00	0.00	0.26	0.09

**Table 6:** Brick parameters determined during porosimetric analysis.

Bricks Type/Property	Unit	Historical Bricks	Factory-Made Bricks	Handmade Bricks
Total Pore Area	(m²/g)	0.3	8.14	0.42
Median Pore Diameter (Volume)	(nm)	6500.6	272.37	3057.7
Bulk Density	(g/mL)	1.81	1.83	1.56
Porosity	(%)	30.75	28.89	32.03

**Table 7:** Historical brick frost resistance prediction models.

Sample/Total Volume of Typical Pore Ranges	Annotation	Historical Brick No. 1	Historical Brick No. 2	Historical Brick No. 3	Average	Classification Frost-Resistant FR/ Not Frost-Resistant NFR
Range from 1.0 to 10 μm [%]	[11]	90,54	89,30	89.28	89.71	FR
Range from 1.4 to 0.25 μm [%]	[18]	16.09	17.92	17.31	17.11	FR
Larger than 3.0 μm [%]	[15,22,30,40,41]	75.14	71.47	73.53	73.38	FR
Larger than 2.0 μm [%]	[19]	83.75	80.96	81.77	82.16	FR
Smaller than 1.4 μm [%]	[18]	16.25	19.04	18.23	17.84	FR
Smaller than 1.0 μm [%]	[19,42]	11.01	13.13	12.88	12.34	FR
Smaller than 0.5 μm [%]	[20]	4.62	6.05	5.87	5.52	FR
Maage factor	[15]	180.45	171.63	176.56	176.21	FR
Franke and Bantrup factor Ф50 [mm]	[43]	7.08	7.03	6.25	6.79	FR

**Table 8:** Factory-made brick frost resistance prediction models.

Sample/Total Volume of Typical Pore Ranges	Annotation	Factory-Made Brick No. 1	Factory-Made Brick No. 2	Factory-Made Brick No. 3	Average	Classification Frost-Resistant FR/ Not Frost-resistant NFR
Range from 1.0 to 10 μm [%]	[11]	2.23	2.92	7.02	4.06	NFR
Range from 1.4 to 0.25 μm [%]	[18]	61.90	65.36	65.57	64.28	NFR
Larger than 3.0 μm [%]	[15,22,30,40,41]	4.12	3.85	6.14	4.70	NFR
Larger than 2.0 μm [%]	[19]	4.33	4.15	6.96	5.15	NFR
Smaller than 1.4 μm [%]	[18]	95.67	95.85	93.04	94.85	NFR
Smaller than 1.0 μm [%]	[19,42]	95.43	95.49	91.74	87.25	NFR
Smaller than 0.5 μm [%]	[20]	93.94	93.02	87.25	91.40	NFR
Maage factor	[15]	10.00	9.35	14.84	11.39	NFR
Franke and Bantrup factor Ф50 [mm]	[43]	0.27	0.26	0.29	0.27	NFR

**Table 9:** Hand-made brick frost resistance prediction models.

Sample/Total Volume of Typical Pore Ranges	Annotation	Handmade Brick No. 1	Handmade Brick No. 2	Handmade Brick No. 3	Average	Classification Frost-Resistant FR/ Not Frost-Resistant NFR
Range from 1.0 to 10 μm [%]	[11]	94.48	85.85	84.05	88.13	FR
Range from 1.4 to 0.25 μm [%]	[18]	0.27	22.24	23.94	15.48	FR
Larger than 3.0 μm [%]	[15,22,30,40,41]	90.11	52.35	59.42	67.29	FR
Larger than 2.0 μm [%]	[19]	99.73	77.76	75.80	84.43	FR
Smaller than 1.4 μm [%]	[18]	0.27	22.24	24.20	15.57	FR
Smaller than 1.0 μm [%]	[19,42]	0.23	16.15	18.72	11.70	FR
Smaller than 0.5 μm [%]	[20]	0.13	7.99	10.30	6.14	FR
Maage factor	[15]	216.40	125.71	142.69	161.59	FR
Franke and Bantrup factor Ф50 [mm]	[43]	3.80	3.03	3.25	3.36	FR

## Data Availability

Not applicable.
